# Single-Virus Microscopy of Biochemical Events in Viral Entry

**DOI:** 10.1021/jacsau.4c00992

**Published:** 2024-12-31

**Authors:** Marcos Cervantes, Steinar Mannsverk, Tobin Hess, Diogo Filipe, Ana Villamil Giraldo, Peter M. Kasson

**Affiliations:** † Department of Biomedical Engineering, University of Virginia, Box 800759, Charlottesville, Virginia 22908, United States; ‡ Science for Life Laboratory and Department of Cell and Molecular Biology, Uppsala University, Box 596, Uppsala 75124, Sweden; § Departments of Biomedical Engineering and Chemistry & Biochemistry, Georgia Institute of Technology, 315 Ferst Dr. NW, Atlanta, Georgia 30332, United States

**Keywords:** single-virus, membrane fusion, fluorescence microscopy, viral entry, microfluidics

## Abstract

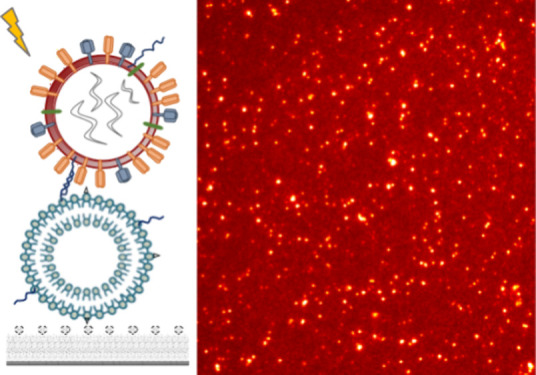

Cell entry by enveloped viruses involves a set of multistep, multivalent interactions between viral and host proteins as well as manipulation of nanoscale membrane mechanics by these interacting partners. A mechanistic understanding of these events has been challenging due to the complex nature of the interactions and the event-to-event heterogeneity involved. Single-virus microscopy has emerged as a powerful technique to probe viral binding and fusion kinetics. Single-event distributions compiled from individual viral particle measurements have enabled estimates of protein stoichiometry at fusion interfaces, a better understanding of the rate-limiting steps for fusion, and a more robust identification of the biochemical regulatory factors for viral entry. Recent technical advances have made these experiments feasible on less specialized microscopes, increasing their accessibility to a broad range of scientists. Single-virus entry kinetics have now been measured for a wide range of enveloped viruses and on both synthetic and physiological substrates. Here, we briefly review the major progress in the area. We then describe the critical apparatus, protocols, analytical techniques, and optimizations needed for robust measurements of virus-membrane interactions.

## Introduction

**Figure 1:**
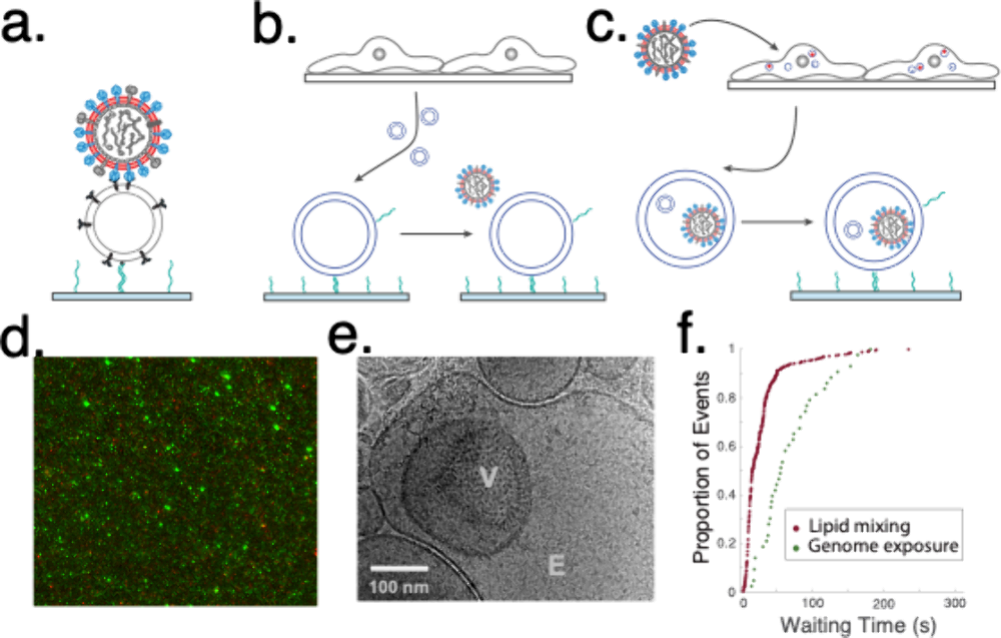
Single-virus fusion. Panels (a)–(c) depict a single-virus fusion to synthetic vesicles, plasma membrane vesicles, and endosomes. Panel (d) shows a fluorescence micrograph of SARS-CoV-2 virus-like particles (red) bound to plasma membrane vesicles (green), panel (e) shows a cryo-electron micrograph of virus inside endosomes (reprinted from ref (1). Copyright American Society of Microbiology), and panel (f) shows comparative kinetics of lipid mixing and nucleic acid exposure through a full fusion pore (data replotted from ref (8)).

In order to successfully infect a host cell, lipid-enveloped viruses must overcome a series of hurdles, including host attachment, fusion protein activation, and fusion of the viral and host membranes. Elegant video microscopy approaches have been developed to monitor these stages of the viral entry process in cells,^2−7^ but controlling the viral and cellular components required for entry is challenging. Performing single-virus microscopy of fusion outside cells yields a simplified, more controllable approach to directly probe the minimum requirements for the early stages of the viral entry process. Such single-virus experiments were initially developed to test fusion between viral particles and synthetic bilayers.^9,10^ A broad range of chemical tools have since been added (Figure 1) not only to a) vary the chemistry of viral entry itself but also to b) control the geometry and composition of target membranes from simple synthetic substrates to intact subcellular membranes.^1,11−16^ Here, we describe the key design elements and protocols of a single-viral fusion assay that has been used extensively to probe mechanistic and biochemical factors in the entry process for influenza, Zika and SARS-CoV-2, and other viruses.^15,17−23^

Membrane fusion by enveloped viruses almost uniformly occurs in the endocytic pathway: at the plasma membrane or in endosomes, phagosomes, or lysosomes.^4,24−29^ Viruses attach to host-cell receptors and undergo fusion protein activation, which results in the insertion of a fusion peptide or fusion loop into the host membrane. These protein–lipid interactions have been aptly reviewed elsewhere^30^ and ultimately result in refolding of the viral fusion proteins and membrane fusion, opening a pore that permits subsequent viral uncoating and entry of the genome into the cytoplasm. Because this process involves complex pathways with heterogeneous outcomes, single-event measurements are often more informative about the mechanism than bulk ones. However, the technical challenges in performing such assays have often proven to be a barrier to use by scientists less familiar with these assays. This paper aims to facilitate the use of single-virus fusion assays by discussing the experimental design considerations and detailing protocols to implement them.

Broadly, single-virus fusion assays require a viral sample, a target membrane, sufficient activation and trigger factors for fusion, a fluorescent probe for viral state changes, and video microscopy to measure the kinetics of single-virus fusion events. We and others utilize microfluidic flow cells to create a controllable environment for fusion. The assay we describe here differs from many other variants in two key respects: a) using vesicles and endosomes as primary target membranes rather than supported lipid bilayers and b) using either endogenous receptors or DNA-lipids as attachment factors. These two choices permit variation of membrane curvature, the use of cellular membranes with native or native-like compositional asymmetry, and the separation of host cell attachment from potential conformational activation of viral proteins by receptor ligation.

Some key findings from implementing this assay have provided insights revealing that (a) influenza binding to receptor functions primarily as an attachment factor,^15^ (b) in contrast, ACE2 receptor binding conformationally activates SARS-CoV-2 spike protein but these changes are also thermally accessible in the absence of ACE2,^19^ (c) an off-pathway state exists in Zika fusion that determines pH sensitivity,^17^ and (d) influenza fusion also likely has an off-pathway state, as endosomal lipids modulate the extent of full fusion but do not affect the observed extent of hemifusion or initial lipid mixing.^31^

## Experimental Design

**Figure 2:**
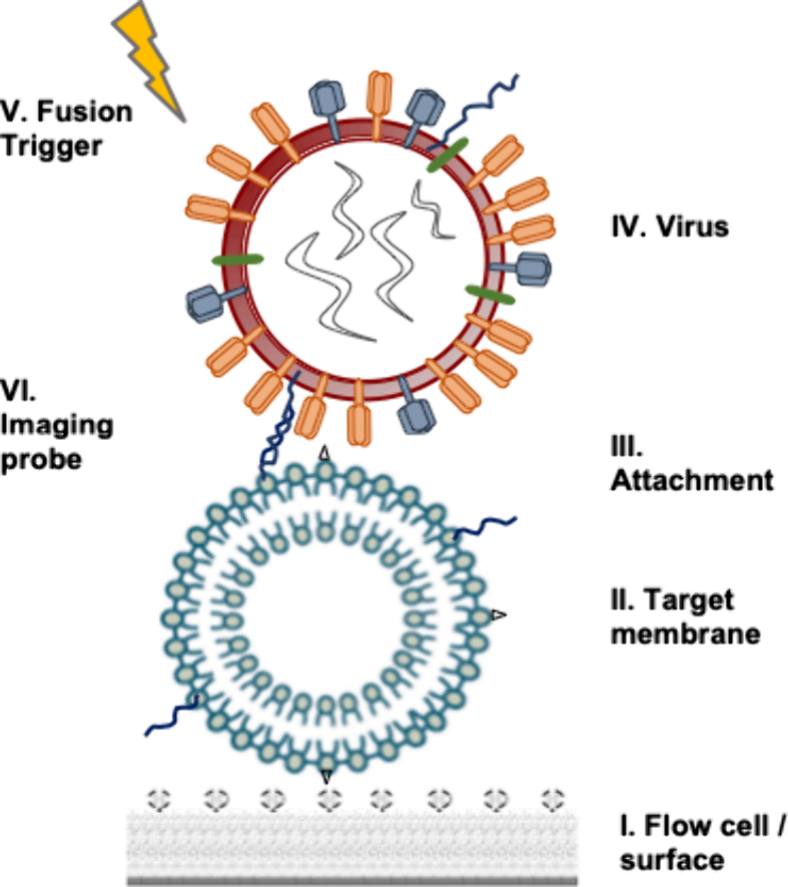
Key components of a single-virus fusion assay. This schema depicts the arrangement of the six key components discussed in experimental design.

The single-virus fusion assay described here has 6 fundamental components, schematized in Figure 2. Many of these components involve exchangeable elements that can be swapped to examine different viruses, synthetic vs physiological target membranes, and the like. We explain the fundamental design considerations behind these elements and then present a detailed protocol for such a set of choices. Swappable elements are contained within green boxes, and details for alternative protocols are provided in the Supporting Information.

### Flow Cell/Surface

Soft lithography^32,33^ is used to form parallel-channel microfluidic flow cells of PDMS bonded to a glass coverslip via plasma activation. This design permits multiple channels to be functionalized and target membranes to be prepared in parallel, although our typical practice is to bind viruses, trigger fusion, and image sequentially. Channel-to-channel variability in observed single-event statistics is much lower than flow-cell-to-flow-cell variability, so higher parallelism within a flow cell permits more precise comparisons between conditions. The final considerations are surface functionalization and passivation. We typically functionalize with a base layer of PLL–PEG-Biotin followed by an avidin coating but have also used N_3_ surface functionalization followed by addition of alkyne-ssDNA to yield ssDNA-functionalized surfaces. Surface passivation and blocking are important concerns to reduce nonspecific binding, which can reduce reproducibility. In addition to passivating any remaining reactive groups, we typically block using BSA or binding-incompetent liposomes.

### Target Membrane

This assay permits a broad choice of target membranes: synthetic liposomes,^15^ membrane-functionalized nanoparticles,^14^ plasma membrane vesicles,^18^ and even harvested endosomes with entrapped virus^1^ have all been used in our single-virus fusion configuration. The assay design also permits choice of target membrane geometry, including planar bilayers, small unilamellar vesicles and similar-sized objects, or giant plasma membrane vesicles. Each can be the appropriate choice for certain experiments: we prefer small vesicles tethered to the flow-cell surface because those have a lower concern for defects and better protein mobility than planar bilayers while retaining a relatively planar imaging characteristic. In contrast, giant vesicles extend farther upward in the flow channel, creating more three-dimensional imaging conditions (and also potentially less uniform flow conditions). We typically use synthetic membranes when tight control over lipid composition is desired and when endogenous nucleic acids will interfere with imaging probes. We use membrane-functionalized nanoparticles when tight control over the membrane curvature is desired. We use plasma membrane vesicles when cellular lipid composition and some degree of cellular lipid asymmetry are desired and also when endogenous receptors are desired without reconstitution. Finally, we use virus-endosome conjugates when endosomal composition and geometry are required, with the virus fusing to the interior surface of the endosome.

### Viral Attachment

Viral attachment to target membranes can be achieved either using endogenous receptors in native or reconstituted form or via DNA-lipid tethers;^15,34^ each has its own distinct advantages. Endogenous receptors recapitulate physiological conditions more closely, yet they can pose challenges with reconstitution. Some of these challenges include: the choice of proper receptor when there is not yet broad agreement nor a single identified receptor, as well as the effect of variations in receptor expression on plasma membrane vesicles. DNA-lipid tethers provide a synthetic means of attachment yet are explicitly designed only to attach viruses while not conformationally activating the spike protein. As such, additional factors are required for viruses in which receptor engagement normally provides a conformational signal for spike activation. In the case of DNA-lipid tethers, one ssDNA-lipid is added to the target membranes at low concentration, while a complementary ssDNA-lipid is incubated at low concentrations with the viral sample. DNA hybridization then mediates attachment.

### Viral Particles

This assay can be used with infectious viruses, virus-like particles, or pseudovirus, depending on the desired biosafety profile and virological goals. We have performed this assay using infectious influenza A^15^ and Zika virus,^17^ HIV pseudoviruses displaying a broad variety of spike proteins,^18,19^ and SARS-CoV-2 virus-like particles.^35^ Others have also extended it to paramyxoviruses,^21^ and slight variations on the assay have been used for an extremely broad range of enveloped viruses.^23,36−40^ For most assays, we label viral particles with Texas Red-DHPE to permit the visualization of bound particles and measurement of lipid mixing. Many different dyes can be used; we selected Texas Red based on data showing photochemical effects with some dyes that can impact the fusion process.^41^ The lipid dye can be omitted entirely if a different fluorescent probe for fusion is desired.

### Fusion Trigger

For fusion to occur, the appropriate physiological fusion triggers must be provided. For many viruses, such as influenza and Zika, this is simply a change in pH, which can be accomplished via a buffer exchange in the flow cell. In cases where receptor/coreceptor engagement provides the fusion trigger, this can be accomplished either upon viral binding to a target membrane with appropriate (co)receptors or by addition of soluble receptors *in trans* when viral attachment is achieved using DNA-lipid tethers. Finally, in cases where proteolysis provides the final trigger, either soluble proteases can be added to the flow cell, or membrane-bound proteases can be used. In the case of SARS-CoV-2, we also found that viral incubation with protease before introduction to the flow cell surprisingly yielded robust fusion.^18^ Video microscopy should begin shortly before the fusion trigger is introduced: either after viral binding if the trigger is a soluble factor added later, or at the time of viral introduction if receptor engagement or membrane-bound proteases provide the trigger.

### Imaging Probe for Viral State Changes

Real-time probes for single-virus state changes provide the assay readout. In the simplest version of this assay, viral lipid mixing can be measured via dequenching of a fluorescent dye in the viral membrane. Viral membranes are preferred over target membranes for reasons of relative abundance and signal-to-noise. However, we have also used dequenching of calcein dye^20,31^ or alternately a nucleic-acid-binding dye, each loaded into the target vesicle lumen,^8^ to report on fusion pore formation. Additional probes can be added for immunolabeling of specific proteins or quantitation of target membrane area, enabling individual binding and fusion events to be selected on the basis of such criteria.^1,8,14^ Such immunolabeling probes can also be used posthoc to image viral antigens if desired.

## Materials

### Reagents (Standard Lab Reagents Are Omitted)

- Kapton polyimide tape (Ted Pella)
- Glass microscope slide (25 × 75 mm, 1.0 mm thickness)
- Polydimethylsiloxane SYLGARD 184 Silicone Elastomer Kit
- Glass coverslips (24 × 40 mm, #1.5 thickness)
- 7X cleaning solution (MP Biomedicals)
- PLL–PEG (SuSoS)
- PLL–PEG-Biotin (SuSoS)
- Reaction buffer (10 mM Sodium Phosphate Monobasic, 90 mM Sodium Citrate and 150 mM Sodium Chloride, pH 7.4 or 5.0 as indicated)
- Neutravidin (Fisher Scientific)
- Palmitoyl oleoylphosphatidylcholine (Avanti)
- Dioleoylphosphatidylethanolamine (Avanti)
- Cholesterol (Sigma)
- GD1a (Sigma)
- DHPE-Biotin (Avanti)
- DiYO-1 (AAT Bioquest)
- Virus of interest (X-31 H3N2 influenza is used as an example here)
- Texas Red-DHPE (Fisher Scientific)
- HEPES buffer (20 mM HEPES, 150 mM NaCl, buffered to pH 7.2)
- PD SpinTrap G-25 (Cytiva)
- Ceramic coverslip rack
- Scalpel
- 2 mm biopsy hole punch
- 2 mm tubing
- 1 mL Luer-lock syringe

### Equipment

- Laboratory furnace (≥400 °C)
- Glow discharger or plasma cleaner (e.g., PELCO easiGlow glow discharger or Harrick plasma cleaner)
- Liposome extruder (Avestin)
- Polycarbonate membrane filter, pore size 100 nm (Avestin)
- High-speed centrifuge (capable of 20,000 RCF or ultracentrifuge)
- Syringe pump with withdrawal mode
- Epifluorescence microscope equipped with temperature control, sCMOS camera, LED illumination (e.g., Andor Zyla, Lumencor Spectra-X, Zeiss AxioObserver with incubation chamber)
- Objective heater (optional)

### Procedure

#### Flow Cell Assembly (Typically Performed over 3 days)

**Figure 3:**
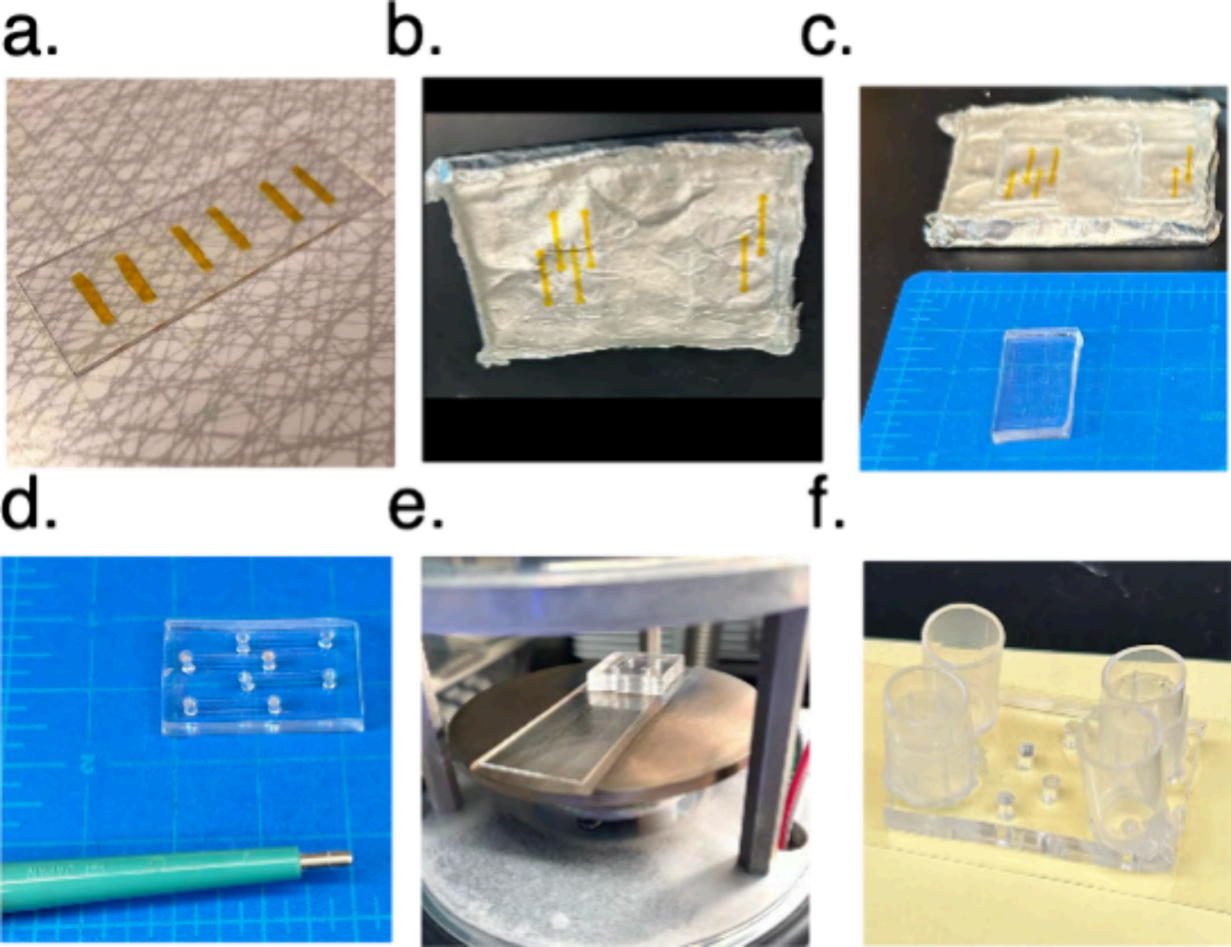
Flow cell assembly. (a) Tape-based lithography for flow cell master molds. (b) Precast PDMS flow cell in master mold. This master shows one two-channel design and one four-channel design, with tape cut using a cutter-plotter (see Supporting Information). (c) A square is cut and lifted out from the PDMS flow cell master mold, containing four tape-based channels. (d) One hole in each end of a PDMS flow cell channel is punched so that each channel has two holes. (e) The cleaned glass coverslip and cut out PDMS square are placed in the glow discharger for plasma functionalization of surfaces. Note that the PDMS flow cell channels should be face-up. (f) Complete assembled flow cell. Each channel contains two holes, where one hole has a buffer well attached to it (cut 1000 μL pipet tip glued to PDMS).

The single virus assay primarily takes the form of a microfluidic flow cell, and these devices offer a great deal of customizability in terms of flow channel layout (Figure 3f). The flow cells are all formed in the same manner; via plasma bonding of a thin glass coverslip to a precast PDMS mold. Following this, the flow cell channel surface is coated in PLL–PEG-Biotin followed by neutravidin, which permits immobilization of the target membrane vesicles and consequent binding of viral particles to the target vesicles.

#### Tape-Based Lithography Master Mold (Time ∼ 2 h)

Master molds for casting the PDMS body of flow cells are made using a tape-based lithography approach. Our tape-based channels have dimensions of 2.0 × 12.5 mm. The master molds can be formed once and reused many times for as long as the master remains intact (Figure 3a). Here we describe the simplest approach using a scalpel; designs for flow cells with more parallel channels using a cutter-plotter are given in the Supplement. After the mold is formed, PDMS is poured into it in the next step to create the actual flow cells (Video S1).

1. Apply Kapton polyimide tape (Ted Pella) without creases over a 25 × 75 mm glass micro slide, with 1.0 mm thickness.
2. Place a stencil of the flow channels (see Supplement) and cut the flow channels from the tape, carefully peeling away the excess.
3. Place the resulting master slide with tape-based flow channels in a 10 cm Petri dish for casting.

#### Mixing and Pouring PDMS (Time ∼ 4 h)

Here we prepare silicone-based flow cells in our prepared master molds. The Kapton polyimide tape cutouts on the glass microscope slides will form the flow cell channels in the casted flow cells.

1. Mix polydimethylsiloxane (PDMS, Sylgard 184) at a 10:1 weight/weight ratio of elastomer: curing agent.
2. Pour the mixture into 50 mL centrifuge tubes and spin at 500 RCF for approximately 5 min to remove bubbles. Then transfer the mixture into master molds and degas under house vacuum for ∼20 min. Reintroduction of atmospheric pressure after this will pop most remaining bubbles. Any residual bubbles can be removed by repeating degassing or dragging the bubbles away from the channels with a clean utensil.
3. Cure the cast polymer at 60 °C for 3 h and allow it to cool (Figure 3b). Alternatively, the molds can be left overnight at 37 °C to cure.

#### Cleaning Glass Coverslips (Time ∼ 7 h)

Here, we describe the process for appropriately cleaning glass coverslips. The working principle is that the glass surface should be as clean as possible prior to plasma exposure and surface functionalization. While other methods that use harsh reagents exist (such as piranha solution cleaning), we prefer cleaning with a gentler and less hazardous, though more time-consuming, detergent-based solution.

1. Place 22 × 40 mm glass coverslips with #1.5 thickness in a ceramic slide holder, which is placed into a beaker along with a magnetic stir bar. Fill the beaker with 7x cleaning solution (MP Biomedicals) diluted 1:5 in DI water. The solution should be turbid.
2. Leave the beaker slowly stirring at ∼75 °C until the detergent solution turns completely transparent (∼20 min). The solution should not be brought close to boiling.
3. Remove the beaker from the heat and allow it to cool slightly before rinsing off the detergent with several excess volumes of DI water. We typically flush continuously for at least 2 h.
4. Place the ceramic holder with slides in a furnace and anneal at 400 °C for 4 h.
5. Store the cleaned glass coverslips protected from dust in a closed container, and only handle them with clean tweezers.

#### PDMS Flow Cell Assembly (Time ∼ 2.5 h)

With precast PDMS molds and cleaned glass coverslips prepared, the flow cells can now be assembled. After functionalization by plasma cleaning, the glass and PDMS bind irreversibly upon contact.

1. Cut and lift out an appropriately sized PDMS chip from the mold (prepared in Mixing and Pouring PDMS), using a scalpel (Figure 3c).
2. With the channel-side facing upward, punch holes in each end of each channel with a 2 mm diameter biopsy hole punch to form inlet and outlet wells (Figure 3d).
3. Remove any residual dust from the channels with a piece of Scotch tape.
4. For functionalization of the PDMS square and glass coverslip, place a cleaned glass coverslip (prepared in Cleaning Glass Coverslips) and the PDMS chip, with the channels facing upward, into the sample chamber of a glow discharger or plasma cleaner (Figure 3e). The degree of cleaning can strongly affect PDMS adherence; settings optimized for our particular Pelco EasiGlow are as follows:
  - Pressure: 0.40 mbar
  - Charge: 15–20 mA
  - Glow: 30–60 s
  - Polarity: positive
  ***Tip:** We find that the optimal settings for functionalization of the PDMS and glass surface depend on lab environmental factors and the state of the plasma cleaning instrument. The settings might require some optimization to achieve stable bonding.*
5. Carefully place the functionalized glass coverslip on a flat surface; avoid touching the functionalized surface. Flip the PDMS chip so that the channel-side will contact the glass and drop the PDMS chiponto the glass coverslip. Observe that the PDMS attaches completely (see Video S2). Gently press down on the PDMS to aid the final attachment to the glass coverslip.
  ***Crucial point:** If not done correctly, the channel may not be completely sealed on the glass, causing the flow cell to be leaky and rendering it useless. We recommend gripping the PDMS square from the sides and dropping it onto the glass coverslip from a height of ∼3 cm. Gravity should give it enough force to disperse onto the glass coverslip efficiently. This may require some practice. If the PDMS can easily be removed after binding or the PDMS corners are not attached properly, it may be that the plasma settings require further optimization.*
6. Add 5 μL of a solution containing 1 mg/mL PLL–PEG and 50 μg/mL PLL–PEG-Biotin in pH 7.4 reaction buffer to each channel. You should see the liquid immediately disperse into the channel once dispensed (see Video S3).
  ***Crucial point:** for all subsequent steps, the flow cell channel must not draw in air bubbles or dry out under any circumstances, as this will disrupt the channel surface.*
7. Cut the top of a 1000 μL pipet tip to create an inlet reservoir for the flow cell. Dip the smooth uncut end of the reservoir in epoxy and attach to the PDMS chip, so that one of the channel wells is within the reservoir (Figure 3f). This is the inlet well for the channel. Avoid occluding the inlet well with any epoxy.
8. Incubate the flow cell 60 min at room temperature or until epoxy has completely hardened.
9. Wash the flow cell channel with pH 7.4 reaction buffer. To do this, fill the reservoir to the brim with buffer, then aspirate from the opposite outlet well with a 1000 μL pipet tip (see Video S4). Remember to leave some buffer in the buffer well to avoid accidentally drawing air into the channel!
  ***Tip**: the pipet tip should be pressed with with gentle force into the outlet well, so that a seal is formed. If you experience air bubbles entering the pipet while aspirating, try to cut off ∼2 mm of the pipet tip. The larger opening of the cut 1000 pipet μL tip will seal the outlet well better.*
10. Remove all storage buffer from the reservoir and add 5 μL of 0.2 mg/mL neutravidin in pH 7.4 reaction buffer in one channel hole, mix and circulate the solution by pipetting from the outlet to the inlet well multiple times (see Video S5).
11. Incubate flow cell at room temperature for 30 min.
12. Wash unbound Neutravidin with the pH 7.4 reaction buffer (see Video S4).
  ***Tip**: The flow cells can now be stored at 4 °C for up to 2 weeks. Keep the buffer wells topped up, to avoid channel drying due to evaporation. We recommend storing the flow cells in a closed box containing water to reduce evaporation.*

### Target Membrane Vesicles (Time ∼ 2 days)

Our microfluidic flow cell based single-virus assay relies on intact vesicles as the target membranes for viral fusion, in contrast to other assays, where supported lipid bilayers are typically used. We have had success immobilizing pure synthetic liposomes, purified endosomes, and plasma membrane-derived vesicles (PMV) in our flow cells. The crucial point is that the target vesicles contain a biotinylated lipid in their membrane for attachment to the neutravidin-coated surface of the flow cell channel.

1. Mix lipid stocks in a glass test tube in a molar ratio of 67% palmitoyl oleoylphosphatidylcholine: 20% dioleoylphosphatidylethanolamine: 10% cholesterol: 2% GD1a: 1% DHPE-Biotin and dry under nitrogen gas. Resuspend the dried lipids in chloroform, and then redry under nitrogen gas. Wrap the test tube in foil to shield it from light and leave it in a vacuum chamber overnight.
2. Resuspend the lipids in pH 7.4 reaction buffer, mix by vortexing, transfer to a plastic tube, quickly freeze and thaw 5 times using liquid nitrogen and 60 °C water, and extrude using 21 passes through an Avestin LipoSoFast extruder using a membrane with 100 nm diameter pores. Collect the suspended liposomes in a new plastic microcentrifuge tube and store at 4 °C with a shelf life of no more than 2 weeks.
3. Remove the storage buffer from the buffer well. Then add 5–10 μL of the vesicle sample to the flow cell channel, followed by mixing and circulating the solution multiple times (see Video S5).
4. Incubate the flow cell containing the vesicle sample overnight at 4 °C to yield a good coverage of vesicles on the channel surface. An alternate procedure is to incubate for 1 h at 37 °C.
5. The following day, wash away unbound vesicles with an excess of pH 7.4 reaction buffer (see Video S4).

### Viral Sample and Fluorescent Labeling (Time ∼ 1 Day)

The single-virus fusion assay primarily relies on the quenching behavior of fluorescent reporter dyes to detect viral fusion intermediates. We most typically label the viral membrane with a lipophilic dye that undergoes dilutional dequenching upon lipid mixing, which is interpreted as viral hemifusion. Alternative strategies permit incorporation of hydrophilic dye in the target membrane lumen to report on fusion pore formation via either dilutional dequenching (calcein) or fluorescence enhancement upon binding to viral nucleic acid (DiYO-1). Both strategies can also be employed simultaneously with dual-camera acquisition and a dichroic beamsplitter.

A number of enveloped viruses have been studied in this manner; here, we describe procedures optimized for influenza X-31, H3N2 A/Aichi/68, which has been sucrose-purified and is at approximately 10^9^ EID_50_/mL stock suspension. Influenza virus is handled under BSL-2 conditions with respiratory precautions following institutionally approved procedures. The protocol described here use Texas Red-DHPE to detect lipid mixing; alternative probes for viral state changes are described in the Supporting Information.

#### Viral Membrane Labeling with Texas Red-DHPE

1. To label viral particles, first dilute a stock solution of Texas Red-DHPE 0.75 mg/mL in absolute ethanol into HEPES buffer (to match the viral stock) at 37 °C. The amount of fluorophore required to achieve dilutional dequenching upon lipid mixing varies with the viral species and concentration and must therefore be optimized. We recommend testing 2, 4, or 6 μL of Texas Red-DHPE and 240 μL of 37 °C buffer.
2. Combine 72 μL of the diluted Texas Red solution with 18 μL of purified virus and gently agitate on a rocker for 2 h at room temperature, protected from light. The virus:dye ratio is a key parameter here. A more extensive comparison of dye labeling quantities and dye photochemistry effects was presented previously.^41^
3. Dilute the labeling mix at least 13 times with buffer (excess buffer is important to help separate free dye) and centrifuge at 20,000 RCF for 1.5 to 2 h at 4 °C. This should pellet most viral particles, and the supernatant will retain excess dye that was not incorporated. For viruses such as Zika where 20,000 RCF is not sufficient for pelleting, ultracentrifugation at 100,000 RCF through a 20% sucrose cushion for 3 h will also suffice.^17^
4. Discard the supernatant and gently resuspend the viral pellet in 18 μL of buffer. For this resuspension step, we find either gently pipetting the pellet or placing the tube on a gentle rocker overnight at 4 °C are equally effective.
5. Either use labeled viral particles or flash freeze and store at −80 °C until use.

### Attachment (Time ∼ 10 min, Longer with Alternative Approaches)

Viral particles must bind to target membranes via an attachment factor. This can be the binding of a model or endogenous receptor by the viral glycoproteins or use of synthetic DNA tethers. The primary protocol is for influenza attachment to glycosphingolipid receptors, where fusion does not begin until triggering, and where a relatively short binding time is sufficient.

#### Glycolipid Receptor

The simplest approach for influenza viral fusion is binding to glycospohingolipid receptors incorporated in synthetic vesicles. During liposome generation we typically include 2 mol % GD1a, so that the liposomes contain sialic acid receptor on their surface.

1. Add 5 μL of the virus suspension to the flow cell inlet reservoir
2. Using a micropipette, withdraw solution from the outlet hole and reintroduce to the inlet to circulate the suspension (see Video S5). Make sure the buffer well is empty before adding the virus. Circulate the viral suspension at least 5x.
3. Incubate the viral suspension in the flow cell for ∼5 min at room temperature. This will promote viral particle binding to the receptor. For influenza, this circulation protocol results in more efficient binding than simple introduction and incubation. An alternative approach is to incubate for 30 min without circulation.
4. Wash away unbound virus using an excess of reaction buffer (see Video S4).

### Fusion Trigger and Microscopy (2 h Warmup Followed by ∼10–20 min Per Video, Depending on Acquisition Time)

The flow cell should contain immobilized vesicles and can contain viral particles bound at this point. For most of our purposes, we perform acquisition with a 100X, 1.49 NA oil-immersion objective.

**Figure 4:**
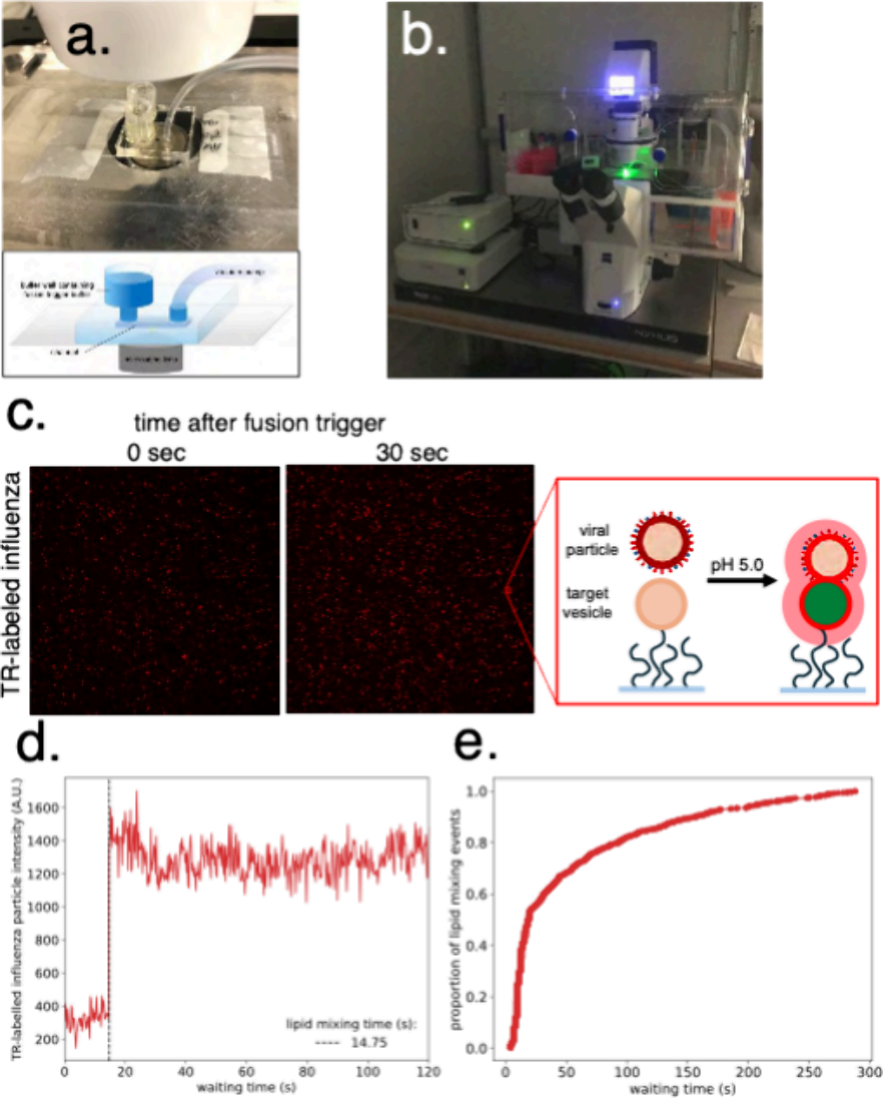
Microscopy and single-virus detection of fusion events. (a) Flow cell attached to microscope holder with 2 mm tubing inserted in the channel hole for buffer exchange. Inset below shows a schematic of a single channel of the flow cell with labels. (b) Image of a microscope with flow cell setup during image acquisition. (c) Typical image before and 30 s after fusion is triggered by buffer exchange. Note that more Texas Red-labeled viral particles are visible after 30 s, as expected from dilutional dequenching upon fusion. Each individual red dot signifies a TR-labeled influenza viral particle, as illustrated by the cartoon on the right. (d) Fluorescence intensity is plotted versus time for a single viral particle with a dequenching spike apparent upon lipid mixing. The interval from buffer exchange (time = 0) to the dequenching spike (black vertical dashed line) is recorded as the single-event waiting time. (e) Waiting times for all individual fusion events are compiled into a cumulative distribution function. The shape of this distribution contains information about the underlying kinetic process and can be analyzed using gamma functions or randomness-parameter calculations.

1. If single-virus fusion experiments are to be conducted at physiologic temperatures, preheat the environmental chamber and objective heater. Allow enough time for all components inside the environmental chamber to reach the desired temperature (∼2 h).
2. Securely attach the flow cell to the microscope slide holder, preventing any movement caused by the syringe pump during image acquisition. We simply tape our flow cell to the slide holder (Figure 4a).
3. Carefully insert a 2 mm diameter tube attached to a syringe pump into the flow cell channel outlet hole (Figure 4a).
  ***Tip**: The tube must be inserted such that it does not slip out during image acquisition. This requires some force and can easily crack the glass coverslip. We recommend keeping your thumb on the underside of the flow cell to counter this force while inserting the tube. This may require some practice and patience.*
4. Ensure that buffer can be withdrawn from the flow cell
5. Scan the flow cell, adjust focus, and locate a suitable field-of-view for imaging. There should be a sizable number of punctate spots in the viral label channel without overcrowding (Figure 4b).
  ***Crucial point**: After focusing on the flow cell channel, we recommend allowing the flow cell to temperature-equilibrate for ∼10 min. We also recommend placing the fusion buffer inside the environmental chamber to similarly equilibrate prior to loading. Temperature fluctuations during image acquisition result in image defocusing, which can be incorrectly identified as fusion events or blur the image so that fusion events are missed. It is crucial to let every component of the fusion setup equilibrate to the environmental chamber temperature. We have found that an objective heater greatly improves thermal stability and reduces defocusing events.*
6. Prepare image acquisition settings. For this sample procedure, we recommend:
  - Exposure time of 150 ms, 4% nominal power on a 260 mW LED (our prior work demonstrates that photodamage from overexposure can inhibit fusion^41^). Measured power at the microscope stage is 900 μW for Texas Red excitation.
  - Frame rate of 4 s^–1^, 1000 frames collected. This ultimately depends on the expected time scale of fusion. The frame rate adjusted to prevent photodamage if longer imaging courses are required. This is a balance between reducing photobleaching and temporal resolution. Photoinactivation of viruses, particularly when a fluorophore is present, has been described previously in work by us and others.^41−45^ We have found Texas Red more robust to such effects than other dyes such as R18, although by no means immune. Close monitoring and minimization of photon dose is critical.
  - Texas Red excitation and emission filters
7. Set syringe pump to withdraw at least 200 μL at ≥30 μL/min but do not activate.
  **Triggering Fusion with Low pH.** This is the most straightforward method of triggering fusion and works for viruses which are triggered by endosomal pH. The protocol given here is for human influenza A, which fuses well at pH 5.0. This is achieved by buffer exchange inside the flow cell, which occurs rapidly upon activation of the microfluidic syringe pump.
  (1) Fill the inlet well with at least 300 μL of reaction buffer, pH 5.0 (excess buffer to prevent bubbles).
8. Start video microscopy, activate the syringe pump, and note the video frame number when flow begins, as this will be time 0 in subsequent intensity time-traces. Buffer exchange (Video S6) will trigger viral fusion inside the flow cell chamber and fluorescence events will be observed over time (Figure 4c).
  Soluble factors such as pH equilibrate across the flow cell on a 1–2 s time scale. For fusion processes exhibiting rapid overall kinetics, this can lead to a corresponding 1–2 s ambiguity in the placement of time 0. If this introduces a substantial error in fusion kinetics, it can be eliminated using a pH-sensitive fluorophore such as Oregon Green^15^ or fluorescein^9^ to monitor the time of pH change *in situ*.
9. Readjust image focus now and as needed during remainder of video time series.
10. After image acquisition, save the video as an image stack file.

### Single-Virus Event Analysis

The goal of video micrograph analysis is to extract time-traces of the fluorescence intensity corresponding to each single-virus spot (or target membrane reporter bound to a virus in the case of alternative fluorescent probes). These can then be assembled into an estimate of the lipid- or content-mixing efficiency and a cumulative distribution function of waiting times from the fusion trigger to the measured fusion event. Subsequent kinetic analysis utilizes these single-event waiting times and distribution functions. We employ a semiautomated analysis pipeline^15^ (https://github.com/kassonlab/micrograph-spot-analysis) that performs image segmentation via thresholding and background subtraction to draw regions of interest around well-defined Gaussian-approximated spots. This pipeline employs heuristics to label spots likely to be single viral particles and reject ones likely to be aggregates; these heuristics can be modified or overridden if the imaging parameters are substantially different. The integrated background-subtracted intensity value of each region of interest is then plotted against time. Particles that exhibit a characteristic dequenching pattern (Figure 4d) are classified as fusing; an interface is also provided to manually review and revise these classifications if desired. The time points of these events are recorded to create empirical cumulative distribution functions (Figure 4e) and estimates of the fraction of particles undergoing fusion (fusion “efficiency”). Subsequent analysis functions create bootstrapped confidence intervals for fusion kinetics, nonparametric statistical testing to compare distributions, and analysis of multistep reactions using randomness parameters^46−48^ and gamma-function fits.^9^

#### Lipid Mixing Kinetic analysis

This waiting time is recorded for each individual particle that underwent such an event and plotted as a cumulative distribution function of all waiting times, usually normalized to one (Figure 4e). This curve reports on the fusion kinetics of the viral particles and can be compared to those of other viruses or conditions.

1. Using MATLAB, execute the Run_Me_To_Start.m script in “Extract Traces From Video” to process video micrographs and extract single-particle time traces.
2. Execute the Start_Trace_Analysis_Program.m script in “lipid mixing analysis scripts” to perform initial scoring of single-particle time traces for fusion.
3. Execute the Start_User_Review.m script in “”User Review Traces” to perform user review of the initial scores.
4. Execute the Start_Fit_Multiple_CDF.m script in “Data Fitting” to fit and compare fusion waiting times between one or more sets of conditions.

## Conclusion

Recent technical advances have made it possible to measure individual viral fusion events to synthetic membranes, plasma membrane vesicles, and even within endosomes. Although technically more demanding, these single-virus approaches have made it possible to probe the reaction stoichiometry, systematically vary receptors and triggering factors in a fashion not possible within living cells, and further elucidate the effect of viral and host membrane composition on the entry process. We anticipate that further advances will broaden the selection of fluorescent probes available to measure additional changes in the viral fusion process, increase the assay parallelism to permit systematic comparison of viral libraries or host factors, and facilitate correlative approaches with other measurement techniques, such as electron cryomicroscopy. We hope that this in-depth discussion of the protocols and pitfalls in single-virus microscopy will both facilitate application to more biochemical questions in viral entry and help accelerate these technical developments.

## Supporting Information Available

Additional methods for swappable elements in experimental design, including flow cell diagrams (PDF)
Video S1 demonstrating flow cell casting (MOV)
Video S2 demonstrating PDMS placement on coverslip (MOV)
Video S3 demonstrating addition of PLL–PEG to flow cell (MOV)
Video S4 demonstrating flow cell washing (MOV)
Video S5 demonstrating circulation in flow cell (MOV)
Video S6 demonstrating buffer exchange to trigger fusion (MOV)

## Supplementary Material

au4c00992_si_001.pdf

au4c00992_si_002.mov

au4c00992_si_003.mov

au4c00992_si_004.mov

au4c00992_si_005.mov

au4c00992_si_006.mov

au4c00992_si_007.mov
