# Supplementary material for: Single-Virus Microscopy of Biochemical Events in Viral Entry
Source: JACS Au. 2024 Dec 31;5(1):399–407. doi: 10.1021/jacsau.4c00992 (PMC11775682; doi:10.1021/jacsau.4c00992)
Supplement: Supplementary file 1 — au4c00992_si_001.pdf [file au4c00992_si_001.pdf]

Supporting Information for
Single-virus microscopy of biochemical events in viral entry

Marcos Cervantes1, Steinar Mannsverk2, Tobin Hess3, Diogo Filipe2, Ana Villamil2, and Peter M. Kasson1,2,3*

Alternative protocols are given for flow cell construction, target membranes, attachment, triggering, and analysis below.

1. Alternative flow cell construction

Kapton tape can be similarly applied to a glass surface and then cut using a cutter-plotter (Cameo 4, Silhouette) to produce more parallel channel designs. A sample design file is given below.

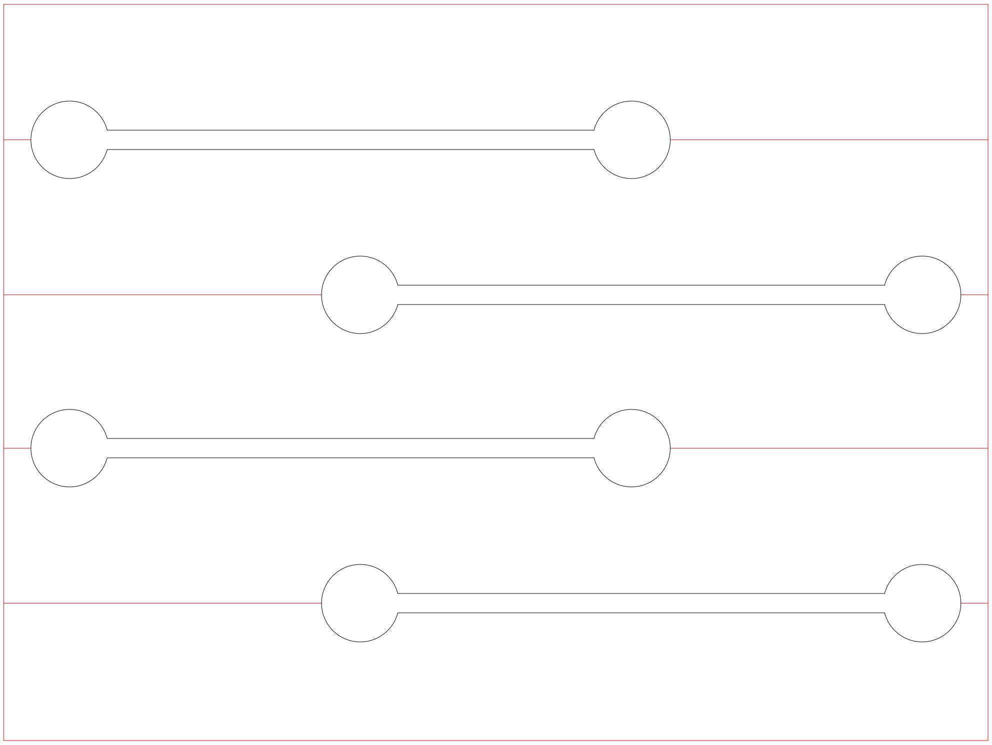

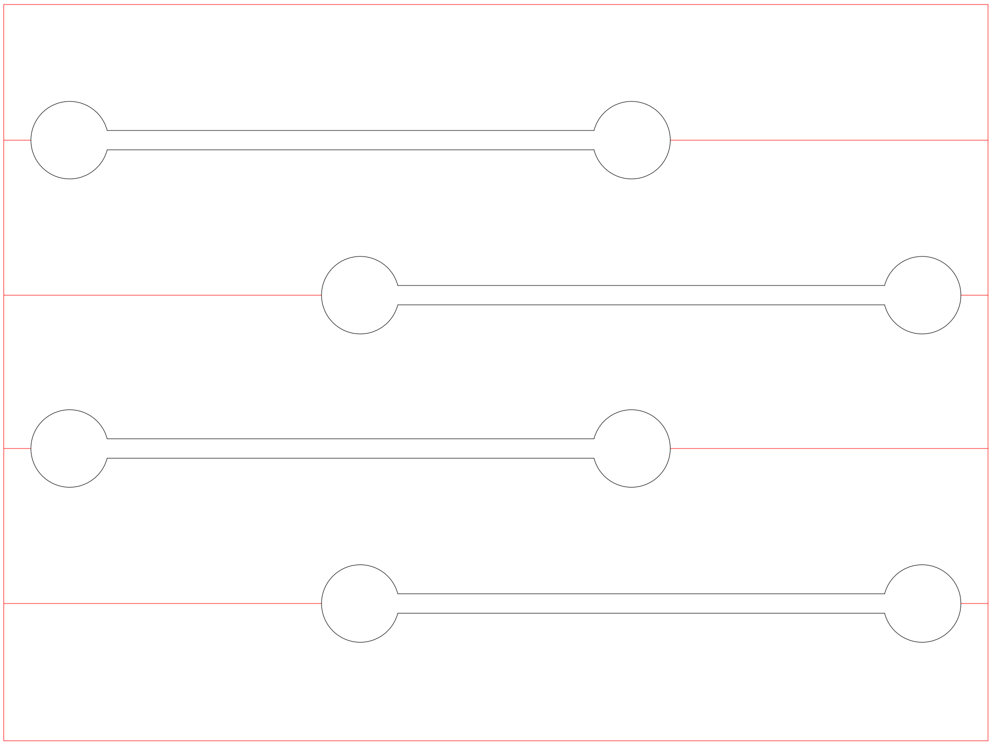

2. Alternative target membranes

Plasma membrane vesicles. We have had success immobilizing plasma membrane vesicles (PMVs) in the flow cell channel. This allows dissecting viral fusion with the plasma membrane, in a near native state. This protocol is adapted from similar prior protocols[1, 2].

1. Grow Vero E6 cells in a 175 cm^2 flask until confluent.
2. Remove media and add 10 µL of DiO in 5 mL of new growth medium.
3. Incubate at 37ºC, 5% CO_2 for 15-20 minutes.
4. Remove excess dye by washing the flask with 5 mL of PBS twice, followed by once with 5 mL of GPMV buffer (150 mM NaCl, 2 mM $CaCl_2$, 20mM HEPES, pH 7.4).
5. Add 5 mL blebbing buffer (GPMV buffer with 0.07% paraformaldehyde, 2 mM DTT) and incubate at 37 ºC for 1-4 hours.
6. Tap the flask gently to aid PMV detachment.
7. Collect supernatant and centrifuge at 150 RCF for 5 minutes to remove cell debris.
8. Transfer supernatant to a new centrifuge tube and centrifuge based on the desired size of PMVs:
   - **Small PMVs.** 36,000-38,000 RCF for 1 hour.
   - **Medium PMVs.** 1,000 RCF for 10 minutes - 1 hour.
9. Discard supernatant and resuspend the pellet containing PMVs in 50-100 µL of pH 7.4 reaction buffer.
10. Add Biotin-DHPE to a final concentration of 50 µM. Care must be taken so that the volume of biotin-DHPE, which is typically dissolved in ethanol, does not exceed 10% of the vesicle sample volume, to avoid disruption of the vesicle membranes.
11. Incubate overnight at 4ºC or place on a gentle rocker for 2 hours at room temperature to incorporate the Biotin-DHPE.
12. Use immediately or store at -80 ºC.

3. Alternative labeling approaches:

Nucleic-acid sensitive dye loading. To report on viral genome exposure, which we also classify as content mixing, we have had recent success through incorporating a nucleic-acid sensitive dye, DiYO-1, inside the target liposomes[3]. Upon pore formation between the viral particle and target liposomes, DiYO-1 binding to the viral nucleic acids results in a >100-fold increase in fluorescence quantum yield. An advantage of this dye is that we are directly detecting exposure of the viral genome, instead of relying on non-specific dye dequenching. However, a disadvantage is that the target membrane vesicles must be RNA-free and DNA-free, which limits this application to synthetic liposomes.

1. After nitrogen effusion and vacuum drying lipids, the lipids are rehydrated in a buffer containing 10 µM DiYO-1.
2. After extrusion of these liposomes, excess extravesicular DiYO-1 is removed by a G-25 spin column.

4. Alternative attachment approaches

Endogenous viral receptor. For cell-derived vesicles like plasma membrane vesicles, the viral receptor is usually naturally expressed on the plasma membrane so that viruses will automatically bind the target vesicles in the flow cell. In cases where (co-)receptor binding can itself trigger fusion, video microscopy should be performed during introduction of the virus, and single-event waiting times are recorded as the interval between binding and fusion.

Receptor-free DNA-lipid tethering. Alternatively, we have also employed the use of synthetic attachment factors in the form of DNA-lipid tethers[4]. These complementary pairs of oligonucleotides are conjugated with a lipid tail and are effective in attaching virus particles to target liposomes without engaging receptor-binding domains of viral glycoproteins as we have previously reported[4-6].

1. Add 1 µL of 6 µM DNA lipid to 100 µL target membrane (liposomes or PMVs) containing between 10^{11} and 10^{12} particles/ml.
2. Add 1 µL of 1 µM DNA lipid to 15 µL of Texas Red labelled virus or pseudovirus containing between 10^8 and 10^9 particles/ml. Complementary DNA strands are used for target membranes and virus or pseudovirus samples to achieve receptor-independent binding.

5. Alternative triggering approaches

Triggering fusion with heat treatment. This method has been used for studying HIV entry[7-9]. Fusion is triggered by increasing the temperature to 37 ºC after allowing viral particles to bind at 4 ºC to cell-derived target membranes that express endogenous receptor and co-receptor.

Triggering fusion with protease. Similar to triggering with an acidic buffer, fusion can be triggered with a buffer containing protease for certain viruses[10]. In a similar fashion, this is added after labeled viral particles have been bound to target membranes.

Alternatively, this protease buffer can be pre-incubated with viral particles before attachment to target membranes occurs. The pre-treated viral particles can subsequently be diluted, added to the flow cell reservoirs, and withdrawn under flux during image acquisition. This method allows for probing factors related to more complex mechanisms of action at the expense of a slightly more complicated variation of the general experiment.

6. Alternative analysis approaches

Lipid mixing efficiency analysis. The total number of dequenching events divided by the total number of bound viral particles is also measured as the lipid mixing efficiency. This should be analyzed with care, as vesicle coverage, non-specific viral particle binding and fluorescently labeled artifacts may affect the denominator in this quantity. Careful controls and comparisons performed across channels within a single flow cell increase accuracy. Where content mixing is measured, the analogous quantity is the number of content mixing

events divided by the total number of fluorescently labeled viral particles. We have also measured content mixing with non-labeled viral particles, in which we use immunofluorescence to label the viral particles *in situ* post-fusion trigger.

References

(1) Sezgin, E.; Kaiser, H. J.; Baumgart, T.; Schwille, P.; Simons, K.; Levental, I. Elucidating membrane structure and protein behavior using giant plasma membrane vesicles. *Nature protocols* **2012**, *7* (6), 1042-1051. DOI: 10.1038/nprot.2012.059.

(2) Levental, K. R.; Levental, I. Isolation of giant plasma membrane vesicles for evaluation of plasma membrane structure and protein partitioning. *Methods Mol Biol* **2015**, *1232*, 65-77. DOI: 10.1007/978-1-4939-1752-5_6 From NLM Medline.

(3) Villamil Giraldo, A. M.; Mannsverk, S.; Kasson, P. M. Measuring single-virus fusion kinetics using an assay for nucleic acid exposure. *Biophysical Journal* **2022**. DOI: 10.1016/j.bpj.2022.11.002 (acccessed 2022/12/05).

(4) Rawle, R. J.; Boxer, S. G.; Kasson, P. M. Disentangling Viral Membrane Fusion from Receptor Binding Using Synthetic DNA-Lipid Conjugates. *Biophys J* **2016**, *111* (1), 123-131. DOI: 10.1016/j.bpj.2016.05.048.

(5) Webster, E. R.; Liu, K. N.; Rawle, R. J.; Boxer, S. G. Modulating the Influenza A Virus-Target Membrane Fusion Interface With Synthetic DNA-Lipid Receptors. *Langmuir* **2022**, *38* (7), 2354-2362. DOI: 10.1021/acs.langmuir.1c03247 From NLM Medline.

(6) Cervantes, M.; Hess, T.; Morbioli, G. G.; Sengar, A.; Kasson, P. M. The ACE-2 receptor accelerates but is not biochemically required for SARS-CoV-2 membrane fusion. *Chemical Science* **2023**, *14*, 6997-7004. DOI: 10.1039/D2SC06967A.

(7) Melikyan, G. B.; Markosyan, R. M.; Hemmati, H.; Delmedico, M. K.; Lambert, D. M.; Cohen, F. S. Evidence That the Transition of HIV-1 Gp41 into a Six-Helix Bundle, Not the Bundle Configuration, Induces Membrane Fusion. *Journal of Cell Biology* **2000**, *151* (2), 413-424. DOI: 10.1083/jcb.151.2.413 (acccessed 2/9/2022).

(8) Frey, S.; Marsh, M.; Gunther, S.; Pelchen-Matthews, A.; Stephens, P.; Ortlepp, S.; Stegmann, T. Temperature dependence of cell-cell fusion induced by the envelope glycoprotein of human immunodeficiency virus type 1. *J Virol* **1995**, *69* (3), 1462-1472. DOI: 10.1128/JVI.69.3.1462-1472.1995 From NLM Medline.

(9) Henderson, H. I.; Hope, T. J. The temperature arrested intermediate of virus-cell fusion is a functional step in HIV infection. *Virol J* **2006**, *3*, 36. DOI: 10.1186/1743-422X-3-36 From NLM Medline.

(10) Sengar, A.; Cervantes, M.; Bondalapati, S. T.; Hess, T.; Kasson, P. M. Single-virus fusion measurements reveal multiple mechanistically equivalent pathways for SARS-CoV-2 entry. *Journal of Virology* **2023**, e01992-01922. DOI: 10.1128/jvi.01992-22.
